# Water scarcity in agriculture: An overview of causes, impacts and approaches for reducing the risks

**DOI:** 10.1016/j.heliyon.2023.e18507

**Published:** 2023-07-21

**Authors:** Carlo Ingrao, Rossana Strippoli, Giovanni Lagioia, Donald Huisingh

**Affiliations:** a Department of Economics, Management and Business Law, University of Bari Aldo Moro, Italy; b Institute for a Secure and Sustainable Environment, University of Tennessee, Knoxville, TN, USA

**Keywords:** Human population growth, Water scarcity, Water footprint, Environmental and social-economic effects, Mitigation solutions

## Abstract

Freshwater is a vital resource for both ecosystem health and human survival, and it is the natural resource that is the most extracted at the global level. Excessive freshwater consumption can be responsible for a scarcity in the circulation rate, which occurs when the freshwater demand exceeds its availability. Hence, water consumption needs to be optimised in all human activities, given the increasing freshwater scarcity due to climate changes and to the annual net increase in the human population of 81,000,000. Freshwater plays many important roles in daily life for example, agriculture is responsible for nearly 70% of that withdrawal volume, and it is therefore, the most water-intensive sector. This puts emphasis upon the urgent need of transitioning towards more sustainable agricultural and food-production/consumption systems. Water Footprint (WF) is increasingly playing a guiding role in that context. Indeed, it makes it possible to quantify water consumption and related environmental consequences. With the objective of contributing to enhancement of research and of supporting practitioners and decision-makers in environmentally sustainable and resilient food production/consumption, the authors of this article addressed the relevant issues connected with: a) physical and economic water scarcity in agriculture, b) practices and tools to reduce water wastage, c) WF assessment methodologies. A number of environmental, economic, and engineering solutions were proposed to mitigate water scarcity. The improvement of irrigation technologies and practices was identified as an important major way to reduce water scarcity. Additionally, solar powered ‘reverse-osmosis’ is being used in many parts of the world to produce irrigation water from saline water, thereby reducing the need to extract freshwater from underground aquifers. This article confirmed the importance of research on water scarcity; moreover, it can stimulate development and application of solutions that make agricultural production/consumption more efficient and resilient.

## Highlights

• A comprehensive review of the literature on Water Scarcity (WS) was carried out.
• The causes and exposure levels, and the resulting impacts were reviewed.
• WS-index assessment methods were also reviewed, recommending plurality in their use.
• WS was found to be responsible for grave impacts on the planet’s health.
• A set of socioeconomic, environmental, and engineering solutions were found.

## 1 Introduction

Freshwater is a vital abiotic resource for ecosystem health and human survival on this planet, as it is essential for people’s lives, agriculture, and manufacturing processes [1,2].

Freshwater is renewed through the water cycle, but excessive consumption can cause shortages in the supply [3]. In many countries the human health and well-being and natural ecosystems are being seriously affected by the changes in the global water cycle, that is due to climate change and to environmentally-harmful and water-intensive human activities [4]. Therefore, considering the current water usage patterns, it is no longer adequate to consider water as a renewable natural resource [5].

Most of the freshwater human use is withdrawn from groundwater, although its turnover rates can vary substantially from one aquifer to another, and within the same aquifer [6].

Bayart et al. [1] identified the following key issues that pertain to freshwater consumption with critical environmental and socioeconomic repercussions:

- the resource depletion, that is due to extraction surpassing regeneration over the long-term, with the risk that the resource may be unavailable for future generations; and
- the competition for freshwater that occurs when the supply for certain users makes it less available to others over a limited time span, with consequent problems of appropriate allocation of that resource among a wide array of users.

Freshwater can be regarded as the natural resource that is most extracted from earth, considering the annual withdrawal of over four trillion m^3^; that quantity is due to population growth, rising living standards, and expansion of irrigated agriculture [4,7]. There is an increasing demand for freshwater to produce foods and feeds, and a wide range of other commodities, to support industrial processes, and to sustain urban and rural populations’ needs [8]. The increasing global demand for freshwater, is resulting in groundwater overdraft in all of those regions that are affected by scarcity of surface water [7]. Freshwater scarcity is increasing rapidly in many regions of the globe; it has become one of the crucial and frequently debated concerns from societal survival perspectives [1,8].

This should be attributed to groundwater being pumped, in those regions, at a replenishment-exceeding rate, which contributes to depleting aquifers and rivers: some of the latter run dry for long periods of the year before they reach the sea [8]. This is done to meet the rising anthropogenic demands and to support international trade, with the consequence that freshwater scarcity is increasingly posing serious threats to global sustainability [9]. Irrigation is globally recognised as the major purpose of those demands, as it utilises about 70% of water used in society [10,11].

Freshwater consumption is, one of the main environmental aspects for which agricultural production is responsible, along with land use, fossil fuel consumption, and the emission of Greenhouse Gases (GHGs) and of other polluting compounds, although it contributes to human health and prosperity as well [12].

Global freshwater use – that includes withdrawals for agriculture, industry and municipal uses — has increased from 500 billion m^3^ in 1900 up to nearly 3.9 trillion m^3^ in 2017, due to human population growth at an annual net increase of about 80, 000, 000, and due to economic shifts towards more resource-intensive consumption patterns [13].

Agricultural production is responsible for nearly 70% of that withdrawal volume, while the industrial sector and the domestic uses are responsible for 22% and 8% of that volume, respectively [14]. Therefore, it is clear that agriculture is the most water-intensive sector, thereby contributing extensively to freshwater scarcity [15,16].

The increase in water demand and the degradation of freshwater due to urbanisation, agricultural intensification, and climate changes, have become major-concerns, especially in regions that are already under water stress conditions [17,18]. More than 25% of the world’s population and more than 40% of the global agricultural production, heavily rely upon unsustainable groundwater extraction [7].

This highlights the urgent need for transitioning to more sustainable agricultural systems. The latter are such that the crops are cultivated with reduced abstraction of freshwater resources compared with the conventional ones that are currently being used in some regions [16]. Modified crop cultivation systems need to be implemented based upon strategies for a more environment-friendly and consumer-oriented agriculture [19,20]. From an agricultural-water management perspective, that would be achievable through the adoption of water-conservation oriented practices along with the treatment of wastewater to obtain water useable for irrigation purposes [16]. Additionally, if Water User Associations (WUAs) use Internet of Things (IoT) and precision agriculture solutions that will contribute to mitigating climate change from several perspectives, including water footprint (WF) reductions [21].

Care has to be taken, however, in preserving soil quality, as well as the yield, quality, and safety of agro-foods, while ensuring that future generations will also have water to produce foods, fibres, nutrients, and other bio-based materials to responsibly and equitably satisfy their needs [16].

In this context, net-regenerative, agro-food supply chains and diets that enable freshwater resource preservation, can reduce the risks of water scarcity (WS). The sixth Sustainable Development Goal (SDG) is focussed upon making improvements of water quality, increases in water-use efficiency, and reductions of water degradation and scarcity on the global scale [22,23]. This is clearly connected with the SDG # 2 with the focus upon achieving food security and improved nutrition, and promoting sustainable agriculture.

Additionally, based upon [24] research, the SDG # 6 shares several targets not only with SDG # 2 but also with SDG # 3 (‘*Good health and well-being’*), SDG # 12 (‘*Responsible production and consumption*’), SDG # 13 (‘*Climate action’*), and with SDG # 15 (‘*Life on land’*).

Progress in that direction can be achieved by shifting to Mediterranean diets which contribute to achieving those goals, by providing not only the potential health benefits in terms of energy, fibre, and nutrients content, but also the potential daily reduction of around 750 L of consumptive WF per capita [25]. This emphasises the importance of using tools to assess and monitor water resource utilisation and pollution in agro-food systems and, thereby, reducing the damage to human health, resources, and ecosystem quality, as starting points to finding and implementing sustainable and equitable water scarcity mitigation solutions [4,16].

The WF analysis approach is increasingly playing a guiding role in this context. It makes it possible to quantify water consumption and related environmental repercussions, as the essential starting point for evaluation of feasible improvement potentials through process revisions and streamlining water quantity management [26,27].

Carbon Footprint (CF) and WF analyses and, more generally, the Life Cycle Assessment (LCA), are valid and useful methods for comparison of different products and processes, in order to determine the options that should be used, based upon the magnitude of the environmental impact reduction that they can help society to achieve more sustainable societies [16,26].

The WF is not only about estimating and understanding water use by human activities and its subsequent multidimensional environmental consequences, but it is also interlinked with CF [28]. Both WF and CF are useful environmental indicators that can help decision-makers to implement farming systems to achieve the delicate balance among challenges such as climate change, water availability, water quality, water security, and agricultural production [15,29,30].

It is clear that climate change is one of today’s largest global concerns that is altering precipitation patterns and is making extreme climate events increasingly frequent and severe which are causing extreme periods of droughts and floods [[31], [32], [33]]. Those events affect water availability and water quality, and they reduce the capacity of ecosystems to provide adequate supplies of water to fulfil human needs, including those for food production and processing [28,31].

Therefore, according to Refs. [14,34], research is needed to develop and to implement practices that help to reduce water usage practices which are overly consumptive and degradative^1^ of freshwater in the supply chain steps that primarily contribute to local water scarcity, directly and indirectly.

With the objective of contributing to expanding research in this content area, the authors performed this literature review with the main objective of exploring the state-of-the-art on:

a) the physical and economic causes of water scarcity in agriculture.
b) the exposure levels of water scarcity, and the resulting environmental and socioeconomic impacts;
c) the array of practices and tools that can be/should be implemented to reduce water wastage considering its centrality in the global processes of water consumption and pollution.
d) the WF assessment methods that are focussed upon water scarcity on the mid- and end-point levels.

Attention was focussed upon agriculture, because it has been documented to be the most water-intensive production sector, and it poses the highest risks to water scarcity.

**Fig. 1:**
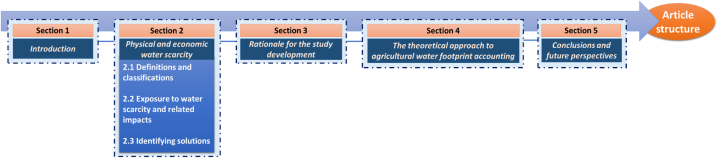
Structure of the study conducted and presented in this article.

Consistently, with this objective and scope, the article was structured as shown in Fig. 1.

This article is part of the authors’ research on WF and on other relevant environmental-performance indicators in the agro-food sector, for production of foods that are less water-demanding, less carbon-emitting and, overall, more environmentally sustainable. This article will help to contribute to accelerating the transition of current agricultural practices to net-regenerative agricultural practices, and to greening the sectors in which those foods are used, including at agro-touristic destinations.

Agro-tourism is defined as the combination of agriculture and rural tourism, and is recognised worldwide as helping to preserve the cultural heritage in rural areas, and to ensure sensorial experiences, authenticity, and seasonal, local foods and drinks, especially when it is coupled with gastronomic tourism paths [35]. Within this perspective, sustainable food tourism is increasingly making the world a better place to live, as it includes innovative, ‘green’, and safe food production at the macro and micro-levels. Additionally, it is based upon a more comprehensive application and a more inclusive understanding of sustainability, and upon transformative food experiences that can increase healthful and ethical food consumption practices [36].

Throughout this article, the authors sought to enhance the reader’s awareness that water consumption needs to be optimised in all human activities, given the increasing freshwater scarcity and the important roles of water in daily life. They also emphasised that studies like this – and like the many others that are expected and welcomed in the future – can contribute to underscoring the importance of making and implementing policies that can support practitioners and decision-makers in the field. Moreover, such studies can help to transform the current patterns of agricultural production and food consumption into more efficient and resilient ones. Such improvements will also help societies to address global challenges, such as climate changes and water availability, through more sustainable, less-intensive, and more carefully-implemented farming and food production and consumption practices.

## 2 The type of review conducted, and its expected contributions to the literature

There are several review articles, which were focussed upon exploring WS-related research. In that context, two highly relevant reviews were published by Refs. [23,37].

Gleick and Cooley [23] reviewed the WS concepts and definitions, metrics and indicators for WS assessments, along with mitigation solutions and, they highlighted the importance of moving forward to more extensive and complete solutions that can help to ensure meeting the basic human and ecological needs for water in the short and long-term future.

Wang’s et al. [37] review was mainly focussed upon the research gaps in physical water stress assessments, which documented to be related to environmental flow requirements and to water pollution. They highlighted the need for standardisation, and harmonisation of terminologies and approaches.

Additionally, some other reviews were focussed upon specific WS-related matters, and/or on specific regional and temporal scales. This was the case of [38], who reviewed water planning-and-management related indicators in Spain, and found that their use can effectively support water management decision-making. Other, relatively-recent, articles that were relevant to this study included those of [[39], [40], [41]]. Together with [23,37]; they deepened the knowledge about the WS subject, including definitions, indicators, accounting methods, and mitigation strategies, and they provided guidance that was valuable for the planning and development of this article.

Liu et al. [39] reviewed WS indicators, mainly based upon population, and the availability and use of freshwater. Although some progress has been made during the past decades for quantification of those indicators, the authors highlighted challenges in WS assessments to be mainly related to adequate green-water incorporation, water quality, globalisation, and virtual water trade [40] systematically reviewed the literature of human-health and water-insecurity related issues in Oregon, and found that effective improvement strategies should include water affordability, community education events, assessments of drought risks due to water losses, and improvements of safe drinking-water compliance.

In another study [41], reviewed the WS status in Sri Lanka and of the water scarcity related causes that contributed to broadening the literature on the subject. They suggested mitigation strategies including: amending/developing legislation; incorporating multidisciplinary assistance; assessing efficiencies in relevant bodies; and increasing awareness of the importance of safeguarding water quality and availability.

In the context of those reviews, this one is the first article designed to summarising the array of important WS-related issues, which puts emphasis upon its novelty and the contribution it can make to this crucial literature. Therefore, the authors’ objective was not to conduct a ‘*systematic review of the literature*’ according to Ref. [42], as that provides a type of scientifically-based, rigorous and in-depth review, that is beyond the scope of this article. Rather, our objective was to perform a literature review, designed to provide an *‘overview’* of the literature on the WS subject.

Therefore, the paper was designed and developed to:

- be easily understood and used by stakeholders in the field of agricultural water scarcity management;
- facilitate knowledge development and dissemination by effectively and clearly communicating the results of the review;
- broaden the body of theoretical work in this content area, which can catalyse future research in this important content area.

During development of this review, the authors focussed upon challenging issues related to information selection and usage criteria:

- *Selection and organisation of data and information*. The authors used generic sources to obtain an overview of the WS-related macro-research area, and, other sources to address particular sub-topics, in depth, such as those regarding the exposure levels and the mitigation solutions. The authors did this to optimise data sorting to ensure that they were thorough in collecting all of the relevant information needed for this review. The authors paid particular attention to the process of evaluating the sources they found, to ensure that the information was valuable and pertinent. Therefore, they used a set of criteria that included accuracy, objectivity, and relevance.
- *Selection bias and a lack of comprehensiveness*. With the objective of circumventing this, the authors designed the search strategy according to literature in ways consistent with the type and aim of their review. They used a set of subject-representative keywords, that included “water scarcity”, alone or in combination with “agriculture”, “impacts” or “effects”, “exposure”, and “mitigation solutions” or “strategies”. To gather the relevant literature, the authors searched in three bibliographical sources, including ‘ScienceDirect’, ‘Scopus’, and ‘Google Scholar’.
- *Using the best data sources*. Source selection was done considering the twofold aim of this review to serve: 1), as the starting base for future original-research articles on the WS subject in agriculture and other linked sectors; and 2), as a comprehensive write-up about the WS topic in general for knowledge enhancement. Consistently, the authors used mainly relatively-new data sources, but looked also at older ones.
- *Lack of a critical approach*. The authors addressed this potential weakness, by conducting their review by a careful evaluation and critical summarisation of the information, in a way that went beyond the simple description of the literature investigated by developing a clear and comprehensive picture of the most important issues of the WS topic.

## 3 Physical and economic water scarcity

### 3.1 Definitions and classifications

Water scarcity is a leading challenge for continued human development, and for the achievement of the SDGs and targets [43]. Water scarcity results from the imbalance between freshwater supply and demand, that occurs when the latter exceeds the former [44,45]. It is a multidimensional, human deprivation state, due to a lack of access to affordable and safe water resources to satisfy their needs, or if their needs are fulfilled but it may be accomplished in ways that cause harmful consequences to the environment in the long-term [45,46].

The current debate on water scarcity is strongly focussed upon the regions approaching the limits of renewable water, where freshwater abstraction and usage are increasingly closer to the total renewable supplies [23].

Water scarcity can affect, entire regions with the results that almost always, the most vulnerable and poor people suffer the most severely from its consequences [45,47]. This underscores the key role that economic and institutional factors play in the process of managing water scarcity. The latter can result from the scarcity of water availability, or scarcity of access because of the failure of institutions that are responsible for ensuring a regular supply or because of the lack of adequate infrastructure [48].

Therefore, when assessing water scarcity, it is the perspective of its physical constraints and the economic determinants that must be taken into consideration [45]. Two types of water scarcity are acknowledged: physical; and economic [49].

The physical water scarcity, also known as ‘*absolute water scarcity’*, which results when demands outpace the limits of regional water-resource availability [49]. Today, about 1.2 billion people (corresponding to one fifth of the world population) live in areas affected by physical scarcity, with the majority of them being arid or semi-arid regions [48].

Physical water scarcity is documented to be seasonal: there are estimates that approximately 67% of the global population live in areas that experience seasonal water scarcities at least one month per year [49]. In this context, it should be underscored that the number of people living under physical water-scarcity conditions will tend to grow dramatically, due to human population growth and to increasing frequency and severity of extreme weather, due to climate changes. [49]. Physical water scarcity affects both of the components that characterise WF, namely the blue and the green ones.

For crop production, green water scarcity (GWS) occurs when there is a rainfall regime that is not able to meet the crop’s water requirements (CWRs) and, therefore, irrigation is needed to prevent crop growth being limited by water shortage [45].

Blue water scarcity (BWS) occurs in GWS-facing croplands, where there is inadequate availability of blue water sources to meet the requirements of irrigation water. Blue water has been at the centre of the water scarcity debate on the global scale, as it underlies the emerging competition that is currently ongoing in the use of water for societal and environmental needs. The BWS is more and more perceived as a global socio-environmental threat, that directly interacts with food and energy security [45,50].

Moreover, target 4 of SDG # 6 explicitly addresses BWS to ensure adequate blue water resources for humankind and for the global ecosystem. Conversely, not enough attention has been given to GWS, and this is surprising if one considers that green water contributes 65% of global crop production [45]. In this context, the worst thing is that, as stated by Rockström and Falkenmark [51], a proper green-water management plan continues to be missing in the SDG agenda.

To be even less explored are economic water scarcity (EWS) related issues, as documented by Ref. [45]. The EWS occurs when blue water is physically available, but there is no economic and institutional capacity to help societies to adequately manage their sustainable usage of that water [45]. This is the situation in Karachi^2^ that, for three decades, has been facing the worst water crisis in its history, not because of problems of natural shortage but due to mismanagement by the planners and managers [52].

According to FAO statistics, more than 1.6 billion people face similar problems of economic water shortages [48]. In areas affected by EWS, although the water would be enough to meet human and environmental needs, there is limited access to it. Mismanagement or underdevelopment may lead to polluting accessible water or to making it unsanitary for human consumption. The EWS may also be the result of the unregulated use of water for agricultural and industrial systems, often at the expense of the general population, and be responsible for inefficiencies in water use, that are often due to undervaluing the economic value of water as a finite natural resource [49].

The EWS is also associated with the condition in which countries have renewable water in adequate quantity and quality to satisfy the current and future water requirements, but need to improve their water development programs and equipment, so that they can sustainably use their freshwater resources [45,53]. In this regard, it is important to note that [45] reviewed the emerging research agendas, including adaptive water governance, and the political-ecology, justice, and community aspects of water and found that those are affecting the relationships between access and restriction, and between possession and dispossession.

This led [45] to conclude that, in order to achieve full understanding of EWS, one needs to consider a variety of socio-political and economic factors that interact at different scales.

From that perspective [45], advanced research in the field by defining the concept of “agricultural economic water scarcity” (AEWS) as the condition in which croplands exposed to GWS are not irrigated, though renewable blue water is sufficiently available for irrigation purposes at the local scale. This lack should be attributed to irrigation being impeded due to a combination of socioeconomic and political factors, such as ‘little attention was given to exploring the details of this phenomenon and its role(s) in the global geography of water scarcity’ [45]. This underscores the need for research in the field to ensure that substantial improvements are made to avoid disruption of irrigation water supplies.

### 3.2 Exposure to water scarcity and related impacts

For this section, the relevant exposure-related issues extrapolated by Ref. [45] were addressed because they help to increase understanding of how AEWS affect water and food security on the global level [45] clarification of the AEWS concept helps to inform policy-makers of the multi-level challenges of water and food security, and can be used to motivate them to urgently work to achieve global sustainability targets.

In their article [45], mapped the GWS, BWS, and EWS indicators across the global croplands of 130 primary crops, in a spatially-explicit and integrated manner. They found that exposure to water scarcity is strongly dependent upon geographic location and the month of the year: they estimated, that GWS is faced by 76% of global croplands for at least one month a year, and by another 42% of those lands for five months a year. The authors estimated that current green water consumption in croplands is 5406 km^3^ per year. Additionally, they found that, to avoid crop growth reductions due to GWS-derived water-stressed conditions, there would be an additional 2860 km^3^ per year of blue water consumption to be required by global croplands [45].

That would mean fulfilling the global water requirements for irrigation without taking due account of the limits imposed by sustainability needs and earth’s carrying capacity [45,54]. Irrigation is increasingly seen as the practice to minimise green water deficits and to boost agricultural production in several regions of the globe, which has made it a crucial factor in global food security.

It should be underscored, however, that this practice is largely exposed to BWS, as highlighted by Ref. [45]. They found that there is a widespread reliance of food production on irrigated regions in BWS conditions. They documented that 68% of the global irrigated croplands go through BWS for one month a year, while 37% of those crops do so for five months. Highly-irrigated regions stay can be found: in the high plains of Unites States and in the Central Valley of California; in Mexico; in Spain; in North China; in the Murray-Darling Basin of Australia; in India; and in Pakistan. Those areas were documented by Ref. [45] to be constantly going through BWS for several months annually.

Rosa et al. [45] expanded their assessment to AEWS and found that it affects 15% (0.14 billion hectares) of global croplands, while 16% of the cultivated lands were irrigated in manners not sustainable. Given that rain-fed production is usually sufficient only one growing season per year [45], found that:

- 0.06 billion-hectare economically water-scarce croplands, that is 43% of the global total, face agricultural EWS for only one month in the course of its rain-fed growing season; and
- 86% of that total (i.e., 0.12 billion hectares) goes through AEWS for three months during its rain-fed growing season.

They documented that AEWS is generally concentrated in low-income countries with large yield gaps. This paper’s authors agree with [45] when stating that should be attributed mainly to the lack of investments in the irrigation infrastructure that is needed to meet CWR by using the available blue water.

As expected, in high-income and arid regions, there are fewer EWS croplands because, in those regions, irrigation expansion may be effective in increasing the production of food and feed. Two-thirds of AEWS croplands are located in Sub-Saharan Africa, in the Eastern part of Europe, and Central Asia [45].

Croplands’ water scarcity results according to Ref. [45] were summarised in Table 1 for croplands with water scarcity less than and more than one month a year.

**Table 1:** Water scarcity distribution in global croplands for time periods less than and greater than one month, based upon [45] results.

Water scarcity type in global croplands	Time period in one year	
	<1 month	>1 month
	Percentage (%)	
GWS	76	42
BWS	68	37
AEWS	43	86

GWS: Green Water Scarcity; BWS: Blue Water Scarcity; AEWS: Agricultural Economic Water Scarcity.

Overall, they concluded that where rainfall is low or access to surface water is limited, reliance on aquifers is a common reality. Over-exploitation of groundwater resources can be threatening for future water supplies if the aquifer-water withdrawal rates exceed the natural recharge ones. This is certainly an issue of global concern, as it is causing distress for one third of the world’s largest aquifer systems [49].

With water resources becoming scarce, problems pertaining to fair and equitable water allocation increase. The result of this may be that governments must make forced choices between agricultural, industrial, municipal, or environmental interests, with the consequence of some groups winning access to water and others not. Chronic water scarcity can result in triggering migration and conflicts both on the domestic- and regional-scale dimensions, especially in those areas that are characterised by geopolitical fragility. In those areas, there is a huge risk for the triggering of water crises, where water supplies decrease to critical levels [49].

In the 2017 Global Risks Report, due to their impact on humanity, water crises were ranked as the third most important global risk, after weapons of mass destruction and extreme weather events [49,55].

**Fig. 2:**
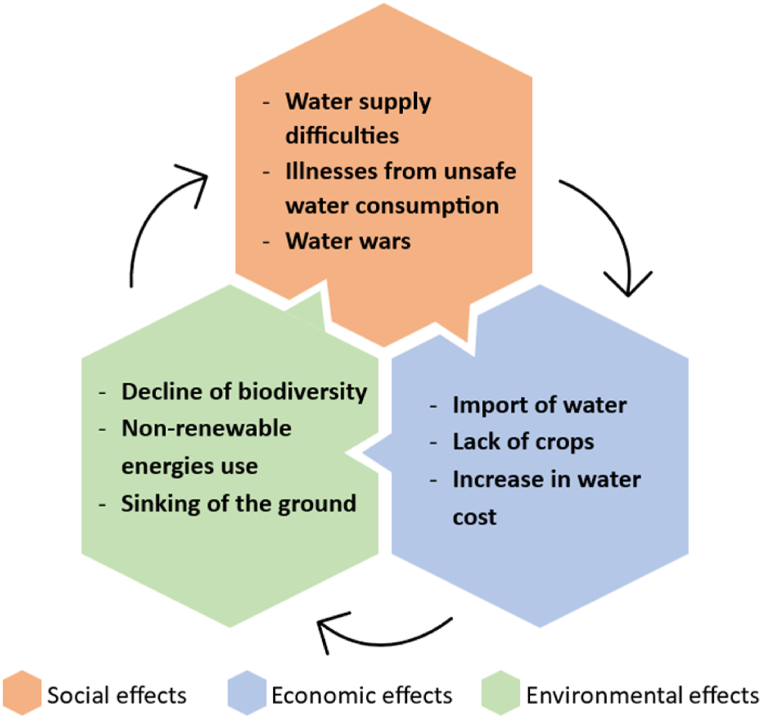
Social, economic, and environmental effects of water scarcity. Source: authors’ elaboration from Study [56].

Overall, water scarcity can have environmental, economic, and social effects: a description of which was based upon the Study [56] publication that was complemented with a synthesis of them in Fig. 2. That was developed to clearly show that those effects are interlinked in a cause-and-effect manner. Among the social effects, it is important to state that shortage conditions can drastically limit the supply of - and therefore, access to - freshwater resources. The solution could be to travel from regions affected by water shortage to those where freshwater is available and accessible. However, that would be inconvenient for many socioeconomic and environmental reasons, therefore it is not practicable.

Therefore, to avoid dehydration, people living in those regions are frequently forced to drink unsafe water at the expense of their health. As a consequence, in many cases they contract typhoid and cholera.

In this context, building dams and reservoirs is often regarded as the most common approaches to cope with droughts and water shortages, as they can store water in the wet periods and release it during the dry seasons. Doing so makes it possible for them to stabilise water availability, and to satisfy water demands and to reduce water shortages [57]. However, dams can lead to conflicts for water supply, as filling dam's reservoirs in one region can temporarily reduce river flows in a bordering region, with the consequence of damaging the entire productive sectors. This is the case of Ethiopia which, by filling its Grand Renaissance Dam, could reduce the Nile’s flows by as much as 25%, thus devastating Egyptian farmlands during the period of filling of the dam.

Also, expanding reservoirs often ends up reducing incentives for preparedness and adaptive actions, which increases the negative impacts derived from water shortages. In addition to this, extended periods of abundant water supply, supported by reservoirs, can increase dependence upon water resources, with the subsequent increase in social vulnerability and economic damage in case of extra severe water shortages [57]. There is evidence that the social and economic effects of water scarcity are highly interconnected. Among the economic effects, when freshwater is not adequate within a country to meet human and ecosystem requirements, there is the need to buy it and have it imported from other countries. To that end, it is necessary to create water supply and collection systems, which implies huge investments must be made to ensure technical and social challenges are duly addressed.

The result of this inevitably leads to higher prices of the water resources. Furthermore, the scarcity of water implies a decrease in available water to irrigate crops, which causes reductions in food production. Because there are many countries that rely upon agriculture to earn money, a lack of crops can to cause them to have financial difficulty and economic decline. Both social and economic effects cause impacts on the environment, such as the destruction of species habitats which results in deterioration of biodiversity. Excessive water extraction can lead to land subsidence that occurs when large amounts of groundwater are withdrawn from groundwater regions of fine-grained sediments.

That is interconnected with the need to treat water to make it drinkable which requires the use of appropriate technologies and much energy. In that context, if the energy used for the cleaning of the water is from non-renewable sources, the climate change related impacts on the environment will increase.

The authors of this article underscore the urgent need for addressing water scarcity through multidisciplinary approaches. From that perspective, Petruzzello [49] recommended the need for proper management of water resources with the goal of equitably optimising economic and social welfare with the uncompromised functioning of the ecosystem and its services. Such an ideal case is often regarded as the “triple bottom line” (i.e., economics, environment, and equity) [49], that are the three dimensions of sustainability, which are addressed in the following section.

### 3.3 Identifying solutions

Several environmental, economic, and engineering solutions have been acknowledged over the years to contribute to reducing water scarcity. They were reviewed by Petruzzello [49] as follows:

Public education is undoubtedly a key factor of water conservation efforts, and additionally, public and environmental policies should be based upon sound science for the implementation of initiatives oriented to sustainable resource management [49].

In this context, according to Petruzzello [49], key strategies to fight against water scarcity are about preserving and restoring natural ecosystems, such as wetlands and forests that facilitate collecting, filtering, storing, and releasing water. That is because, only intact freshwater ecosystems can support ecological processes like nutrient recycling and flood protection, that are acknowledged to have economic and social values [49]. In this context, policies made to incentivise sustainable farming practices were recommended by Petruzzello [49], because they help to protect water sources and the related ecosystems from agricultural pollutants.

Furthermore, Petruzzello [49] proposed to impose water usage taxes on heavy water users, as that could help to reduce wasteful water consumption in industry and agriculture, and to leave household water prices unchanged. The beneficial aspect would be that the tax would ideally help to decouple economic growth from water use, which means that tap water would not belong to the category of products for which consumers would experience higher prices due to the increase in production costs [49].

Those taxes could be assessed to heavy food-waste generators, to stimulate them to reduce food losses and wastes (FLW) and related damage to the environment and contamination of fresh water [58]. In this regard [58], documented that halving global FLW may be effective in reducing global food-production WF by 12–13%.

Moreover, reducing FLW by 50% can make it possible to reduce water scarcity for over 720 million people globally, and to totally eliminate local water scarcity by around 18% of those people [58,59]. This is especially relevant in the context that currently 1.2 billion tonnes of food are lost annually on the farms, and 30–40% of all food marketed from the farms is wasted within the food processing, storage, marketing, and consumption chain [60]. The FLW is a global, multi-dimensional concern, that poses several societal challenges in terms of sustainable development, human health, and the reduction in financial operating costs [61].

**Fig. 3:**
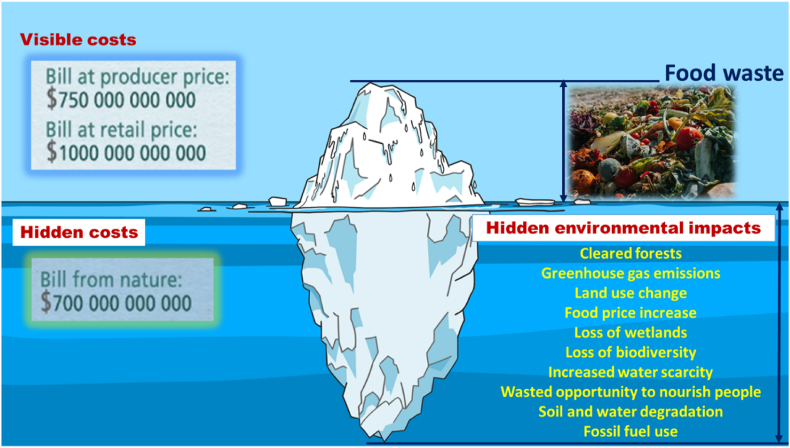
The food waste iceberg: costs and negative economic and environmental effects (authors’ adaptation from FAO [62]).

From that perspective, the contents of Fig. 3, were published in the FAO [62]; which showed that water scarcity and water degradation are environmentally harmful effects from food losses and food wastes that are not properly utilised.

In this context, it is important to implement societal practices of reducing food wastage and of using the ‘wastes’ to make compost for helping farmers to transition to net-regenerative agricultural. Doing so needs to be combined with educating farmers about potential water losses from unsustainable irrigation and farming practices, and about the potential environmental gains resulting from sustainable reduction of food losses [49]. The consumers also need to be educated to rethink their food consumption habits, to avoid wasting food that is expired due to lack of time for its consumption, or that is still edible but is wasted because of the excessive amounts prepared compared with the actual requirements. In other words, it is urgently needed that consumers are educated to avoid over-purchase and over-preparation of foods. In doing so, societies can make progress in implementing SDG#2 and, thereby, contribute to achieving sustainable and less GHG-emitting, less water-intensive agriculture for enhanced food security and improved nutrition.

Theoretically, a combination of governmental incentives and pay-by-weight disposal taxes could help to reduce household food waste, but until now, there is little evidence of that having been put into practice [58,63]. This should be attributed to the lack of research that can guide policy interventions for water scarcity mitigation by means of FLW reduction [58].

Thus far, the research has been primarily focused on quantifying the current FLW in different parts of the food supply chain [58] and not on how to reduce or to make use of the wasted food.

Other effective solutions to reduce water wastage in agriculture could be focused upon: clear water-use reduction targets to be set; and funds to be allocated for water-efficient irrigation technologies [49]. In this context, several water scarcity challenges could be addressed by means of traditional engineering solutions, with the result of immediate benefits. One of the most obvious of those solutions would be to repair the water extraction, processing and delivery infrastructure to reduce water losses in the water distribution networks thereby, avoiding disruption of the water supply services [49].

Additionally, because about 70% of all freshwater resources are used in agriculture, Petruzzello [49] highlighted the improvement of irrigation technologies as a major way to achieve reductions in water scarcity.

In line with this [45], concluded that expansion of usage of sustainable irrigation equipment and irrigation scheduling can contribute to increasing food production without causing terrestrial and aquatic degradation. According to Ref. [45]; sustainable irrigation enables adaptation to climate changes, and creates food production systems that are more reliable and resilient than solely rain-fed croplands [45] estimated that while approximately 810 km^3^ per year of blue water resources could be used for sustainable irrigation worldwide, currently humanity uses 1083 km^3^ per year, with the dramatic consequence of overshooting the planetary boundaries for water.

Researchers have estimated that 0.14–0.23 billion hectares of the aforementioned rain-fed croplands located in the Sub-Saharan Africa, in the East of Europe, and in the centre area of Asia are suitable for sustainable irrigation but, actually, they are not irrigated due to AEWS conditions [45]. Furthermore [45], underscored the urgent need for those AEWS regions to eliminate the currently used, unsustainable irrigation practices and technologies, in favour of a combination of sustainable irrigation deficit practices and irrigation expansion. That would make it possible to provide 13% of global calorie production, meaning producing enough food to feed 1.34 billion people [45].

Another solution for water scarcity can be net-regenerative agriculture, as it helps to develop healthier soils that absorb and hold more moisture from rains and/or from irrigation, which reduces topsoil runoff and reduces freshwater consumption [64,65]. Improving the soil not only leads to a sustainable increase of its fertility, but also improves water infiltration. Enhancing infiltration means reducing runoff, and also reducing erosion and pollution from those soil pieces that are carried away in the runoff water. There are areas, where water springs that dried up several years ago are now flowing again due to new regenerative farming approaches [66]. Regenerative agriculture is based upon five essential principles, that include: minimising the disturbance of soil and the excessive usage of plant fertilisation and protection chemicals; maximising biodiversity of flora and fauna; covering the soil with dedicated crops or residual biomass for as long as possible; and adapting to the local environment. According to Ref. [64]; the common regenerative farming methods include:

- no-tillage system, to reduce heavy digging and ploughing and so preserve the quality and integrity of the soil;
- cover crop use, to enhance soil fertility;
- increasing biodiversity, to increase the variety of nutrients that can go deep into the soil through roots and natural decomposition;
- crop rotation, to make it possible that what it is taken out and put back into the soil by plants is properly balanced;
- combining animals and plants in the same ecosystem; and
- minimising chemical inputs, to reduce impacts on biodiversity and water pollution.

Regenerative agriculture has still some way to go, before it becomes the alternative to the current conventional and large-scale agriculture. For farmers, a regenerative approach would mean using new economic models that are profitable and nature-friendly, and policymakers using alternative ways of considering, developing, and promoting sustainability. Additionally, for ‘change-makers’ working on reducing the negative impacts of farming, it could include an array of small actions and changes that are closely linked to a large-scale vision [67].

Sustainable regenerative agriculture can be complemented with other water-scarcity mitigation solutions like treated wastewater reuse, that is proven to be effective, especially in areas of the world where the human population is growing rapidly and the water supplies are limited [49]. Additionally, reusing wastewater can contribute to improving the quality of streams and lakes as it reduces the polluted effluent discharges. Doing so would make it possible to ease the strain on limited freshwater supplies. [49]. Wastewater can be reclaimed and reused for crop and landscape irrigation, thereby reducing freshwater consumption and resulting in positive environmental and socioeconomic impacts [49]. Doing so would support the implementation of a circular bio-economy and, thereby, would contribute to accelerating the transition towards a sustainable post fossil-carbon society [68,69].

In this context, usage of constructed wetlands is a valuable, clean technology to use for this purpose [54]. Such wetlands are being increasingly used for treating domestic and agricultural wastewaters, mainly due to beneficial features that include environmental quality preservation, landscape conservation, and economic convenience [54].

Additionally, desalination (DS) is a valuable alternative in the fight against WS, as it converts seawater, which has a high sodium content, into potable water, that is suitable for a wide range of purposes and applications [70]. Desalination can be achieved by distillation, electro-dialysis, and by reverse osmosis [70].

Desalted water is being used as a source of municipal water in several, densely-populated arid regions, such as Saudi Arabia. Currently, there are approximately 18,200 desalination plants operating worldwide with a global cumulative capacity of about 90 million m^3^ per day [71]. It is expected that this capacity will be expanded dramatically as a result of increasing fresh-water-shortage crises, caused by climate changes and population growth [71,72].

However, the existing desalination technologies require substantial amounts of energy, usually of fossil origin, is used, which contributes to making the process expensive [49] and is environmentally-harmful due to primary-energy source exploitation, along with the emission of GHGs and other pollutants. That problem can be reduced by using solar powered reverse osmosis systems, as is already being done extensively in some parts of Mexico and in other countries, thereby, avoiding the fossil-fuel related environmental impacts. This approach is a win-win approach, as it has the double benefit of reducing consumption of scarce freshwater and reduces usage of fossil fuel-based energy sources [73].

Additional benefits can be obtained by stopping the disposal of the seawater-desalination derived brines into the sea and using the brine to recover metals such as molybdenum, magnesium, scandium, vanadium, gallium, boron, indium, lithium, and rubidium, which can be a very profitable alternative [71].

Using the brine would make it possible to avoid discharging brine into the sea, which is highly problematic for marine ecosystems due to its high salinity, and to reduce the environmental impacts of virgin metal production [71]. The economic and environmental burdens of desalination depend on various factors, including process configuration, energy source, financial package and the local capital, and operational and maintenance activities [74]. For this reason, it is generally used only in case of economic unavailability of sufficient freshwater.

Desalinated water can be used, along with storm-water, recycled water, transferred water, conserved water, and surface water, for agriculturally managed aquifer recharging (Ag-Mar) [7]. This approach is an emerging water spreading method that aims at transferring excess surface water, during times of excess water availability, onto agricultural land to recharge the groundwater, thereby fighting against water scarcity [7].

From a review of the literature on this subject [7], highlighted that croplands and pastures comprise approximately 40% of the global land surface. Therefore, agricultural land could recharge 200–3200Mm^3^y-1 surplus water into aquifers, by flooding agricultural areas that can be even larger than 500 ha.

There are, however, several challenges and concerns that [7] identified that revolve around agriculturally, managed aquifer recharge, namely:

- crop yield and health;
- groundwater leaching of legacy nitrogen, salts, pathogens, and inorganic contaminants;
- waterlogging of agricultural lands that are adjacent to Ag-MAR sites, with the consequence of leading to hypoxic/anoxic conditions;
- short and long-term effects on in-stream flows;
- economic feasibility, mainly affected by availability of aquifers to be recharged and of the water conveyance equipment and infrastructures;
- water policy barriers; and
- methods for siting suitable Ag-MAR locations.

Other important concerns include effects of this recharge technology on GHG emissions, along with the post Ag-MAR risk of soil compaction and of the worsening of its condition to be travelled over by people, vehicles and farm machinery [7]. Thus far, most of the research using the Ag-MAR approach has been done in California and - to a small extent - in some other countries [7].

In agreement with [7] findings, this article’s authors underscore the need for more R&D on this approach, in order to properly solve those challenges and concerns.

Underground water recharge can be coupled with rainwater harvesting, mainly by digging pits in the ground and using a filtering system, so that clean rainwater is stored and is available when freshwater is scarce [6] documented that for the aquifer area of Nairobi, the average annual recharge is almost 10% of the precipitation, and the rainfall-recharge trends are positively correlated, with increased recharge when rainfall increases.

Rainwater harvesting (RWH) for non-potable functions, such as irrigation, gardening and washing clothes, can effectively contribute to reducing the demand on public freshwater supplies and the strain on storm-water infrastructure [49]. By doing so, RWH can be used as a decentralised technology, for tackling water scarcity in cities and to also reduce urban flooding [75].

The RWH is gaining increasing attention from researchers, worldwide as they consider it to be a viable method for addressing freshwater-scarcity related challenges by contributing to the water supply and run-off mitigation. The actual feasibility of such technologies is often contested by experts in the field, mainly because of the unreliable and inconsistent rainwater supplies, and because the cost is substantially higher than that of centralised systems [75].

**Fig. 4:**
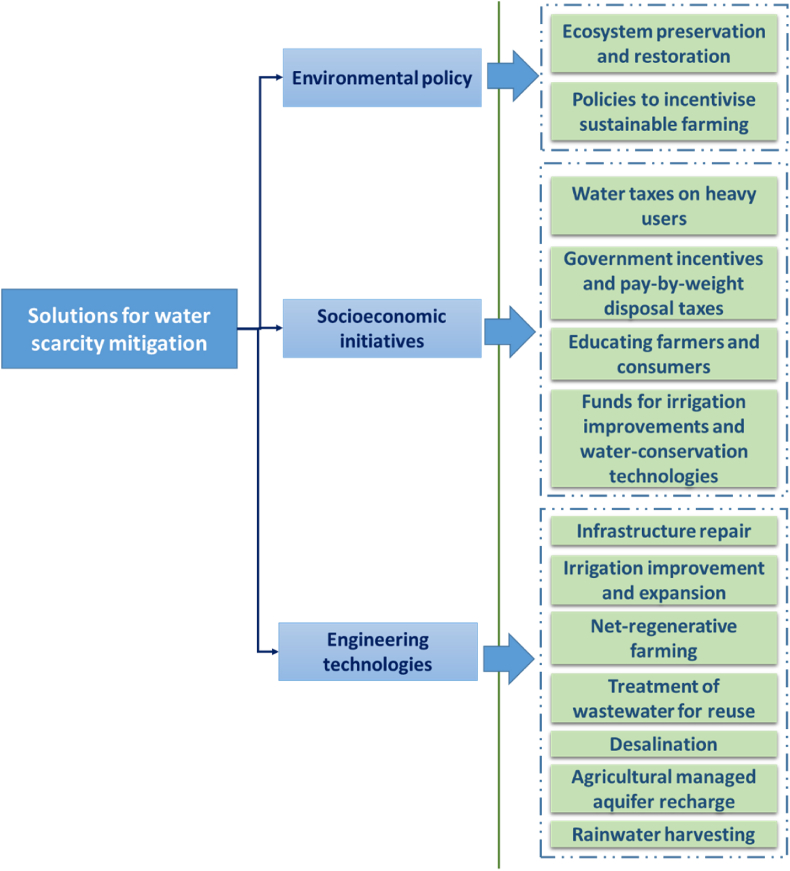
Classification of the main solutions for water scarcity mitigation, based upon [45,49,58,70,75].

In conclusion, according to Petruzzelo [49], the main solutions to contribute to solving the freshwater scarcity problem, from the local to the global scale, were summarised in Fig. 4: a combination of those is recommended as needed for achieving enhanced water-scarcity reduction results in diverse climatological contexts.

## 4 A theoretical approach to agricultural water footprint accounting

The Water Footprint (WF) analysis process helps researchers and practitioners to quantify water consumption and degradation, as the initial step to identify the improvements that can be made and, subsequently, to increase implementation of more sustainable practices for water extraction, usage, and management on the local, regional, and global scale [16,76].

In this context, the European Commission (EU) [77] developed the Water Framework Directive (WFD) to contribute to improving water quality, and increasing availability, and productivity across European Member States [29]. The WF is an aggregate and multidimensional indicator that was developed to respond to the need for quantifying different types of direct and indirect water as a function of space and time [16,78].

As a result [76], developed the WF Assessment (WFA) network (WFN) as a distinct field of research and application on the subject. The WFA evolved with the objective of enabling quantification, interpretation, and reduction of WFs to contribute to sustainability enhancement in all human sectors, including agricultural food production, processing and consumption. The framework addresses freshwater appropriation in a four-step approach, including goals and scope setting, WF accounting, sustainability assessment, and responses formulation [76].

Similar to LCA, the WFA is based upon a detailed inventory dataset of individual processes, and can be classified as a bottom-up approach. Whereas, differently from LCA, which is focussed upon measuring and improving products’ sustainability and, so, can be considered to be a product-oriented method, the WFA was designed to be a water management approach that is focussed upon water resources’ sustainability [37].

The accounting phase of WFA, in particular, is based upon quantifying freshwater usage within three distinct components: the ‘blue,’ the ‘green,’ and the ‘grey’ ones [76]. The ‘blue’ WF indicates the consumption of freshwater that is used in agricultural activities including irrigation, and the dilution of plant fertilisation and pesticidal products [28].

The ‘green’ WF is represented by the crop evapotranspiration, that is only due to the water that remains available in the ground after a rainfall event and that is absorbed by plants to grow [16,28]. In other words, it consists of precipitation and root-zone soil moisture that is available for plant uptake [4,45].

The ‘grey’ WF refers to the water that becomes polluted during a production process and is generally regarded as the water that is required for dilution of pollutants in water discharged into the natural water system, so that the final water quality remains unchanged compared with specific water quality standards [16]. In agricultural WF assessments, ‘grey’ WF is generally increased to the volume of water that is needed for N-fertiliser dilution and that tends to leach into soils and aquifers after it was applied [79].

The total WF is the sum of those three components and refers to the water directly involved in the agricultural processes, while the indirect water, also known as ‘*virtual water’*, represents the water that is embedded in the preparation of the material and energy commodities utilised for the agricultural processes [80].

Each of those WF elements is clearly affected by the way the farming is performed: this is why considering the farmer’s perspective is essential. Farmers and farms are where most food supply chains start. They play key roles in the agricultural water management, as they determine the volumes of:

a. The ‘blue’ water that is withdrawn and applied to the soil through irrigation;
b. The amount of ‘green’ water that is consumed through the ways rainwater is captured by soils and crops, which is strictly dependent upon the types of soils and the farming practices adopted and of course the precipitation of the region;
c. The ‘grey’ water that is generated by water-diluted fertiliser applications [81].

Therefore, farmers must be educated about holistic, net-regenerative agricultural practices of production to be integrated with increased water usage efficiency.

Perhaps, studies like this will help to contribute in this learning process pertaining to the urgent need of changing the current farming patterns towards: water-conservation oriented irrigation solutions; soil quality improvement methods; pesticide and fertiliser use optimisation; and the cultivation of more water-efficient crops based upon the water scarcity of each location [81].

It is clear that global freshwater appropriation is an important issue. In spite of that, there is a global debate on whether WF should be a volumetric or an impact-based indicator as CF is [80]. The debate is rooted on the important need to fully understand and describe the WF purpose, and to determine the most representative unit of measure for water appropriation [80]. This originates from the fact that, while some scientists highlight the importance of impact factors, such as water consumption, is strongly affected by a series of boundary conditions, other scientists argue that a product’s volumetric WF is more important because freshwater is a global resource that is virtually traded through products [82].

Understanding and building upon all of the above perspectives is needed for selection of the WF accounting method, that is most functional and consistent with the aims, scopes, and targets of the assessment study. Currently, two methods can be used, although, they both have methodological challenges [82,83]:

a. The volumetric approach proposed by Ref. [76] based upon the aforementioned WFA methodology for the mapping of a three-component water that is directly and indirectly used along supply chains, which was extensively reviewed by Ref. [29];
b. The impact-based approach that follows the LCA methodology according to the ISO 14046:2014 [84]. This approach explores the cause-effect relationships between water consumption and its environmental repercussions to human health, ecosystem quality, and resources at midpoint or endpoint levels [83].

The ISO 14046:2014 was developed and released to comprehensively address water-related environmental impacts from a life-cycle perspective in a way to encompass water consumption and quality degradation. As a result of this, comprehensive assessments can be conducted that enable creating extensive profiles of impact category indicator results [85]. Some LCA scholars have proposed, that WF should be a tool to supplement the environmental impact assessment of products with a supply-chain or life-cycle approach [80]. Since, ISO 14046 does not recommend a specific impact assessment method to use, several WF-assessment methods have been released over the years [14,34].

In this article, the authors highlighted, in Table 2, the methods that are used to document the water scarcity index (WSI), which is a midpoint indicator, and that are included in the list of WF-assessment methods contained in the Simapro software v. 9.3,^3^ and reviewed by Ref. [83]: they are those of [8,82,86].

**Table 2:** Water scarcity indicator accounting methods in Simapro 9.3.

Method	Approach	Description of the method’s key features
[86]	Midpoint (Water scarcity) Endpoint (Human health)	**-** This WSI-accounting method provides a consumption to availability (CTA) ratio; **-** It was modelled, using a logistic function, so that the resulting indicator values fall within the 0–1 m^3^ deprived/m^3^ consumed. **-** The scarcity indicators considered by this method are available for surface and groundwater. **-** Data on water consumption and availability were extrapolated by using the Water Gap model. **-** The indicator only refers to the volume of consumed water, and assesses the consumptive water use.
[82]	Midpoint (Water scarcity)	**-** This method is based upon the assessment of the vulnerability of basins to freshwater depletion, and measures the water depletion index (WDI) based on local blue water scarcity. **-** The WDI denotes the risk that the water consumption can lead to depletion of freshwater resources. **-** For water scarcity determination, annual water consumption is related to availability in more than eleven thousand basins. **-** The WDI takes into account, lakes and aquifers which had been neglected in previous water scarcity assessments. **-** In addition to water scarcity, absolute freshwater shortage is accounted for by setting the WDI to the highest value in (semi)arid basins. This was used in the method to avoid mathematical artefacts of previous indicators being zero in deserts if the consumption is zero.
[8]	Midpoint (Water scarcity)	- This WSI is based upon a consumption-to-availability ratio. - That ratio is expressed as the fraction between consumed and the available water. - As for [86]; the indicator only applies for the consumed water volume and only assesses consumptive water use.

A description of each method’s key features was taken from Ecoinvent v.3.8,^4^ and was reported in Table 2. Among those [86], is the only method that has been expanded to the endpoint assessment and that, in fact, targets human health, as an endpoint damage category that is affected by water scarcity.

Quinteiro et al. [83] recommended plurality in using all of the current WF assessment methods, as some are complementary in terms of the impact pathway chosen, while others consider different types of freshwater and different forms of usage, as well as different characterisation models. Therefore, the properly-combined use of those methods can provide comprehensive and reliable assessments of WFs of human activities. Their value lies in their capacity to help water planners, managers, and communities in the process of identifying local and community priorities, and for developing strategies that, in the context of sustainability perspectives, help to enable meeting human and ecological needs for water [23]. It also provides insights into where improvements can be made towards increasingly sustainable, bio-based economies.

## 5 Conclusions and future perspectives

This study achieved the proposed goal of reviewing the causes and impacts of water scarcity, as well as highlighting the approaches for reducing the risks related to insufficient water when and where it is needed. This study contributed to understanding the diverse interconnected impacts caused by water scarcity, which are key challenges for achieving the 2030 SDGs and related targets.

In line with this theme, articles by Refs. [23,37]; highlighted that WS is responsible for grave impacts on human health, and upon the planet’s diverse biospheres. The WS poses severe threats to food quantity, quality, and to societal food security.

The authors highlighted, that there exist diverse environmental, economic, and engineering solutions that can help in addressing and solving the WS challenges. The diverse solutions should be used in ways to implement comprehensive, soft-path, approaches, so as to move beyond reliance on traditional supply-side solutions, as suggested by the literature review’ of [23].

If those solutions are not taken in thorough and effective ways, water scarcity will continue to negatively affect the quantities and qualities of food produced and will determine whether there is enough food to feed the human population by 2050. It is a fact that the human population is increasing and may continue to increase in the future: a 9 billion populations in expected by 2050. Population stabilisation is an essential part of the equation that must be addressed along with the others highlighted in this article. That and other issues heighten the urgency for advanced research to develop more, effective water management and human population stabilisation solutions. We must also ensure that there are adequate investments in making these urgently needed changes.

In this regard, it is important to highlight that the World Water Day (WWD) that is held annually on March 22, with the key objective of raising awareness of freshwater importance, and of advocating for sustainable resource management, highlighting the urgent need to comply with SDG #6 and interlinked goals and targets. In 2023 the WWD was focussed upon ways of accelerating change to solve the water and sanitation crises and, because water affects all of humankind, global actions must be taken by stakeholders at all levels, including the domestic ones. Families, communities and societies can make a difference by changing the way they use, consume and manage freshwater in their daily life, especially in those regions living under water stress conditions.

This article contributed to the understanding that there are many of actions that can be taken on the environmental, economic and social dimension, that can be focussed upon improving agricultural water productivity, irrigation efficiency, and domestic and industrial water-use intensity.

In this context, the literature documented the unsustainability of increasing water supply for coping with growing water demand, which is fuelled by the increase in supply. Such approaches put emphasis upon the need for less reliance upon large water infrastructures (i.e., dams and reservoirs), and more reliance upon water conservation measures.

In other words, to cope with drought and water shortage through water consumption reduction, rather than only through water supply improvement, thereby fuelling its excessive consumption, are essential elements of the path forward. From that perspective, sustainable irrigation becomes an increasingly important objective to ensure a reliable and resilient global supply of food in a changing climate on a finite planet called Mother Earth!

The authors wish to highlight that this study was conducted as exploratory for this important and rapidly evolving research field.

They hope that this review’s findings provide valuable inputs for future WF assessments that they and other researchers, will conduct in the sectors of agro-food and related areas such as the sustainable (food) tourism. Doing so, can help societies make progress in the interconnected elements related to climate changes, population stabilisation, geo-political cooperation, and societies beyond wars.

## Author contribution statement

All authors listed have significantly contributed to the development and the writing of this article.

## Data availability statement

No data was used for the research described in the article.

## Declaration of competing interest

The authors declare that they have no known competing financial interests or personal relationships that could have appeared to influence the work reported in this paper.

## Footnotes

1 Consumptive water use refers to the share of water that is removed from available supplies which does not return to a water resource system. The degradative use of water, instead, is regarded as the grey WF component, namely the freshwater that is needed to dilute a contaminant load based upon its concentration under natural conditions and environmental quality parameters [87].

2 With around 15 million people, Karachi is the largest metropolitan area of Pakistan, and is the industrial hub and financial capital of the Region [52].

3 This is one of the most widely used tools that is used for LCA applications [88].

4 Ecoinvent is the world’s most consistent and transparent life cycle inventory database, that supports environmental assessments of products and processes, worldwide [89].
